# Machine Learning-Driven Techno-Economic Uncertainty Analysis in Batch Pharmaceutical Manufacturing

**DOI:** 10.1021/acsomega.5c09455

**Published:** 2026-05-21

**Authors:** Diego Andres Rueda Ordonez, Letícia Costa da Silva Mesquita, Amanda Lemette Teixeira Brandão

**Affiliations:** Department of Chemical and Materials Engineering, 28099 Pontifical Catholic University of Rio de Janeiro, 225 Marquês de São Vicente Street, Gávea, Rio de Janeiro, RJ 22451900, Brasil

## Abstract

Active pharmaceutical ingredients (APIs) are produced in batch multiproduct facilities, where efficient scale-up is crucial for reducing costs and improving productivity. Traditional uncertainty analyses rely on extensive simulations, making them time-consuming and resource-intensive. This study introduces a machine learning (ML)-based framework to estimate techno-economic uncertainties in batch API production, streamlining cost and profitability assessments. Using a pharmaceutical production simulation model for the synthesis of an API via the condensation of quinaldine and hydroquinone, an annual API output of 33,000 kg was estimated, with a baseline unit production cost (UPC) of USD 175/kg. ML predictions showed UPC variations between USD 140/kg and USD 240/kg, impacting the minimum product selling price (MPSP), which ranged from USD 262/kg to USD 525/kg to achieve an internal rate of return (IRR) of at least 30%. An uncertainty analysis revealed that MPSP fluctuates between USD 200/kg and USD 900/kg within a 90% confidence interval. The probability of profitability is only 5% at lower prices but rises to 90% when the MPSP approaches USD 900/kg. The proposed ML framework reduces the computational burden associated with traditional sensitivity analyses by using distinct ML models, including tree-based algorithms, instance-based methods, linear and polynomial regressors, and kernel-based models. This approach provides a fast and effective tool for estimating the UPC and MPSP distributions. With the dataset in hand, the ML-driven techno-economic assessment (TEA) can be used independently of any additional software, making it highly valuable for cost assessment in batch production across pharmaceutical, chemical, and biochemical industries.

## Graphical abstract

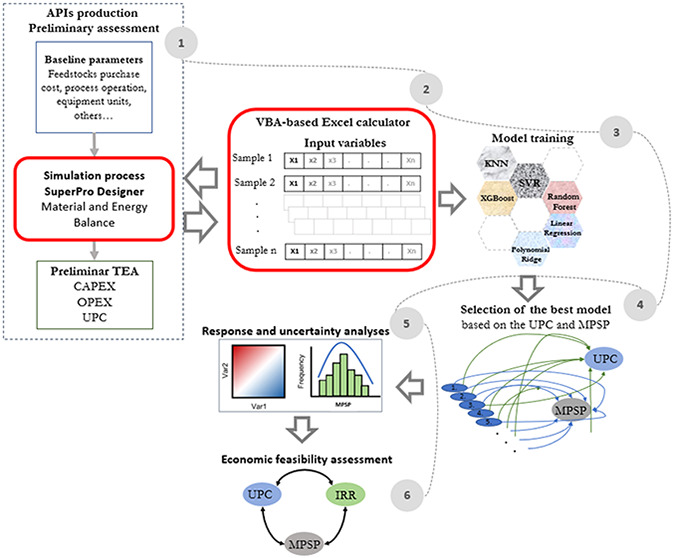

## 1 Introduction

Sociocultural and economic transformations have significantly increased society’s dependence on pharmaceuticals. Key drivers of this trend include accelerated urbanization, industrial expansion, rising per capita income, improved access to healthcare, higher levels of education, and demographic aging.^1^ The pharmaceutical industry has expanded substantially, ranking among the top sectors in the current economic system with global revenues reaching approximately USD 1.6 trillion in 2023, a value comparable to the gross domestic products (GDPs) of major economies such as Spain and Australia.^2^ Although the United States and Europe, particularly Switzerland, Germany, and the United Kingdom, continue to dominate this oligopolistic market, emerging economies (notably the BRICS and MINT nations) are increasingly contributing to the pharmaceutical growth.^3,4^

Amid these economic shifts, the industry’s sustainability and competitiveness are increasingly driven by digital innovations, particularly machine learning (ML) and advanced simulation technologies. Despite this expansion, intensifying global competition and rising demands for affordable medicines have heightened the need for more efficient manufacturing processes. In response, the industry is undergoing a profound digital transformation centered on the use of machine learning to optimize the production of active pharmaceutical ingredients (APIs), streamline research and development, and enhance healthcare delivery.^5,6^ These technologies enable significant cost reductions, accelerate drug development timelines, and improve decision-making across the pharmaceutical value chain.^7,8^

Process modeling and simulation tools, including both commercial and open-source software and custom programming environments, along with scheduling tools, are essential in the pharmaceutical industry, particularly for API production. These tools enable comprehensive modeling of material and energy balances, equipment sizing, labor and utility requirements, cycle times, throughput, and key financial metrics such as production costs, profitability, and cash flow.^9–11^ In addition, they support risk-informed decision-making to inform proactive safety policies, as da Costa et al.^12^ demonstrated through simulation-based fault analysis in a pharmaceutical pilot plant. By dynamically representing process operations, simulations facilitate early identification of inefficiencies, deepen process understanding, enhance communication among stakeholders, and support scenario testing and sensitivity analyses to assess the influence of critical variables on performance outcomes.^13^

While process simulations provide critical operational insights, integrating them with a techno-economic assessment (TEA) enables a deeper understanding of financial viability under varying process conditions. This combined approach strengthens decision-making within smart manufacturing platforms, supporting flexible business models that reduce energy consumption and operating costs while maintaining product quality and process sustainability. TEA adds rigor to economic evaluations by incorporating probability distributions for key financial indicators such as the MPSP, UPC, and IRR, enabling probabilistic assessment of financial viability and investment risk.^14,15^

A reliable TEA framework depends on accurate process models, often developed using specialized simulation software, such as SuperPro Designer. In this study, SuperPro Designer was used to simulate an API production process and establish a baseline economic assessment. This simulation platform, widely used across the pharmaceutical, chemical, and biochemical industries, supports both process simulation and integrated economic analysis.^16,17^

Accurately estimating product cost and profitability is now as essential as ensuring performance, quality, and environmental sustainability. These economic evaluations demand integrated data from across the production chain, from process design to manufacturing.^18^ Knowledge-based methods are thus crucial for formalizing and incorporating this information into digital tools.

Conceptual cost assessments must be rapid and feasible, relying only on data available during the stages of production. However, experimental and pilot-scale validations often entail significant technical and financial resources due to the high cost of raw materials and energy demands, limiting their practicality without specialized expertise.^19^

To overcome these limitations, computational models, ranging from traditional stochastic methods such as Monte Carlo simulations to advanced ML approaches, have become central to cost and revenue prediction in process industries.^20,21^ However, simulations can become computationally intensive when applied to large-scale uncertainty quantification, which has prompted the adoption of ML as a more scalable alternative. There is consequently a growing need for automated frameworks that minimize reliance on iterative simulations while effectively handling input uncertainties and their impact on key techno-economic indicators.^22,23^

ML offers a robust alternative by capturing complex, nonlinear input-output relationships without requiring explicit mathematical formulations.^24^ These models enable faster, more efficient predictions, enhancing decision making under uncertainty and improving the feasibility of TEA.

ML techniques, including artificial neural networks (ANN), support vector machines (SVM), tree-based models (e.g., random forest, decision tree), k-nearest neighbors (KNN), extreme gradient boosting (XGBoost), regression analysis, and evolutionary optimization algorithms, have been successfully applied in API production.^20,22,25^ Unlike traditional models based on deterministic physicochemical laws, ML-driven approaches enhance economic assessments by improving predictive accuracy, enabling efficient policy analysis, and uncovering hidden patterns in large data sets.^26,27^

For instance, Chukhray et al.^28^ developed an ML model using stacking strategies to improve development-stage product readiness assessments and cost estimations, enhancing commercialization efficiency and reducing risks. Kalaichelvan et al.^29^ proposed a fuzzy logic-enhanced ML framework for optimizing pharmaceutical inventory, reducing costs, and improving the supply chain efficiency. Fourkiotis and Tsadiras^30^ applied ARIMA, LSTM, and XGBoost models to forecast pharmaceutical sales using 600,000 historical records, improving trend detection and inventory planning. Additionally, Shiwalni et al.^31^ highlighted how ML is transforming healthcare economics by improving cost-effectiveness, enabling personalized care, and optimizing resource allocation in drug development and telemedicine, despite challenges such as high setup costs, data fragmentation, and privacy concerns.

While ML has been applied to predict process parameters for specific pathways in chemical and pharmaceutical production, research on developing comprehensive frameworks that integrate process simulation, techno-economic analysis, and uncertainty quantification for financial, technical, and market variables remains limited. This gap underscores the potential of ML not only as a predictive tool but also as a core component of integrated decision-support systems, specifically, frameworks that combine ML with simulation and TEA to support decision-making under uncertainty.

Building on recent advances in ML applications for pharmaceutical processes, this study presents an accessible and scalable framework for techno-economic uncertainty analysis applied to a batch API manufacturing process. We present an openly accessible ML-driven approach for analyzing uncertainties in the production of an API synthesized via quinaldine-hydroquinone condensation. Unlike traditional methods requiring extensive simulations to generate large data sets, our framework automatically produces a data set of 5,000 records to train and test ML models. The optimal model was then selected to enable comprehensive economic analysis, estimating key financial metrics while eliminating the need for additional simulations.

To address the high investment risks associated with emerging technologies, the minimum product selling price (MPSP) was evaluated against a 30% minimum attractive rate of return (MARR), as recommended by Mariño et al.^32^ and Tramontina et al.^33^ Economic feasibility was determined by comparing the IRR to the MARR. If the IRR exceeded 30%, the investment was considered economically viable.^34^ Additionally, the framework identifies key cost drivers, such as raw materials, facility-related expenses, labor, and waste treatment costs, enabling a comprehensive sensitivity analysis of their impact on financial performance metrics, including the UPC and MPSP.

The integration of ML into techno-economic analysis offers significant advantages, including the ability to rapidly predict MPSP at any desired scale while accommodating randomized input variables. This capability reduces the time and computational resources required for traditional process simulations while enhancing decision making by providing actionable insights into cost drivers and profitability. Moreover, this methodology can be easily adapted to produce other active pharmaceutical ingredients, further expanding its applicability in the pharmaceutical sector.

## 2 Methodology

This study presents an ML-driven framework for assessing techno-economic uncertainties in the batch manufacturing of an API synthesized via the condensation of quinaldine and hydroquinone.

The API production process was modeled in SuperPro Designer to establish baseline capital (CAPEX) and operating (OPEX) costs. A data set of 5,000 simulations was generated using a VBA-based Excel calculator, incorporating key cost variables such as raw materials, labor, and waste treatment affecting UPC and MPSP.

All programming tasks, including data processing, visualization, and machine learning models implementation, were performed using Python. Multiple machine learning models were trained to simultaneously predict both targets (UPC and MPSP) using scikit-learn’s multioutput regressor, a wrapper class that allows the use of any base regression algorithm by creating a separate regressor instance for each target.^35^ Model performance was evaluated using the coefficient of determination (*R* ^2^), root mean squared error (RMSE), and mean absolute percentage error (MAPE). To further explore economic implications, a two-dimensional cubic interpolation method was applied to examine the joint relationship between MPSP, IRR, and UPC, enabling the generation of a heatmap to visualize revenue fluctuations across scenarios.

### 2.1 The API Manufacturing Process

The process under investigation involves the production of a small-molecule API synthesized through the condensation of quinaldine and hydroquinone. The entire process was simulated using the SuperPro Designer software and is freely available as the SynPharm example on the Intelligen Inc. webpage under the pharmaceuticals section.^16^ This process simulation was previously reported by Petrides et al.^11^

The simulation yields an annual production of 33,000 kg of the API. The baseline plant design assumed an operational lifetime of 15 years, an inflation rate of 4%, a construction period of 30 months, a startup phase of 4 months, a depreciation period of 10 years, and an income tax rate of 40%. Equipment specifications indicated a purchase cost of USD 1,105,000, as detailed in Table S1 (Supporting Information).

The baseline process utilized a batch operation with a scheduling cycle ranging from a minimum of 58.47 h to a maximum of 113.39 h. The facility was designed for an annual operational availability of 7,920 h, supporting the execution of 134 batches per year.

The facility includes three 3,800 L reactors (R-101, R-102, and R-103), two 2.5 m^2^ Nutsche filters (NFD-101 and NFD-102), a 2,000 L storage tank (SB-101), and a 10 m^2^ tray dryer (TDR-101). Although the operating volume is lower (307.5 L), the tank was designed with a 2,000 L maximum capacity to prevent bottlenecks and provide sufficient buffer storage during batch operations. The flowchart process, illustrated in Figure 1, consists of four sections: (i) product synthesis, (ii) isolation and purification, (iii) final purification, and (iv) crystallization and drying. Table S2 presents the raw material consumption for each section.

**Figure 1:**
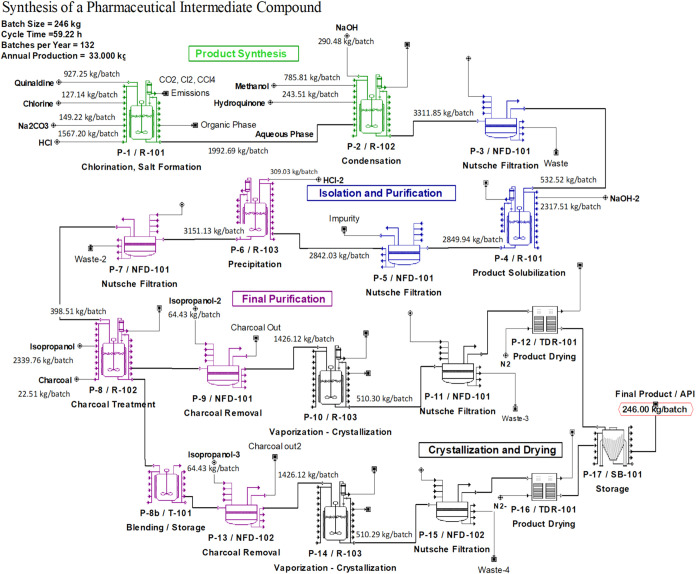
Process flowsheet for API production.

The first step (P-1) takes place in reactor R-101 (P-1/R-101 in Figure 1), where quinaldine is dissolved in carbon tetrachloride (CCl_4_) and reacts with chlorine gas (Cl_2_) to produce chloroquinaldine. HCl is then added to neutralize NaHCO_3_ and convert chloroquinaldine to its salt, a process that requires 16.35 h, as shown in Table 1.

**Table 1:** Stoichiometry and Corresponding Yields of the Reactions Involved in the API Production Process

**stoichiometric reactions of procedure P-1 in R-101**	**yield (%)**
quinaldine + Cl_2_ → chloroquinaldine + HCl	98
Na_2_CO_3_ + HCl → NaHCO_3_ + NaCl	100
NaHCO_3_ + HCl → NaCl + H_2_O + CO_2_	100
chloroquinaldine + HCl → chloroquinaldine.HCl	100

**stoichiometric reactions of procedure P-2 in R-102**	**yield (%)**
chloroquinaldine.HCl + NaOH → NaCl + H_2_O + chloroquinaldine	100
2chloroquinaldine + hydroquinaldine +2NaOH → Impurity +2NaCl + 2H_2_O	2
hydroquinone + NaOH → H_2_O + hydroquinone.Na	100
chloroquinaldine + hydroquinone.Na → product + NaCl	100

**stoichiometric reaction of procedure P-44 in R-101**	**yield (%)**
product + NaOH → H_2_O + product.Na	100

**stoichiometric reactions of procedure P-6 in R-103**	**yield (%)**
HCl + NaOH → H_2_O + NaCl	100
HCl + product.Na → NaCl + product	100

**stoichiometric reaction of procedure P-10/R-103 and P-14/R-103**	**yield (%)**
product → product-crystal	97

In the second step (P-2/R-102), chloroquinaldine.HCl is converted back to chloroquinaldine using NaOH. Hydroquinone, pretreated with NaOH, reacts with chloroquinaldine to yield an intermediate product (Table 1). This reaction lasts 17.63 h and generates impurities that precipitate along with the product. The mixture was filtered using a Nutsche filter (P-3/NFD-101), recovering the solid phase with a 90% yield. Filtration, washing, and cake transfer require 4.44 h.

The resulting cake, containing the product and residual impurities, is dissolved in NaOH (P-4/R-101) to form Product-Na, while impurities remain as solids and are removed by filtration (P-5/NFD-101) with a 95% efficiency. These steps take 17.4 and 5.63 h, respectively. In P-6/R-103, Product-Na is neutralized with HCl over 15.31 h to regenerate the free acid form of the API. Filtration (P-7/NFD-101) recovers the API with a 90% yield in 4.0 h.

In P-8/R-102, the API is dissolved in isopropanol and treated with activated charcoal for 20.17 h to remove chlorate impurities. The solution is then divided for parallel processing, introducing a 15.54-h delay via storage tank P-8b/T-101. Charcoal is removed by filtration (P-9/NFD-101; P-13/NFD-102), which takes 3.51 h.

Crystallization is performed in P-10/R-103 and P-14/R-103, where isopropyl alcohol is evaporated to concentrate the solution. This step yields 97% API over 13.86 h. The crystalline product is recovered via filtration (P-11/NFD-101; P-15/NFD-102), achieving 99% purity in 2.44 h.

Finally, the product is dried in a tray dryer over a period of 15.54 h (P-12 and P-16/TDR-101) and stored (P-17/SB-101). The total process duration ranges from 58.47 to 113.4 h, depending on scheduling.

Notably, the Nutsche filter NFD-101 and the three reactors (R-101, R-102, and R-103) are used repeatedly across different operations. This reuse is enabled by the batch scheduling strategy implemented in the process design.

### 2.2 Techno-Economic Assessment and Dataset Generation

A comprehensive economic evaluation, including profitability analysis and cash flow assessment, was conducted to identify the main factors influencing UPC and the MPSP of the API.

The UPC was calculated as

1 $$\mathrm{UPC}=\frac{\mathrm{OPEX}}{P_{\mathrm{annual}}}$$

where OPEX is the annual operating cost (USD·year^–1^), and *P*_annual_ is the annual production rate of the reference product (kg·year^–1^). Thus, UPC represents the cost per unit mass of product.

The MPSP was determined through discounted cash flow analysis. The selling price was iteratively adjusted until the Net Present Value (NPV) of the project equaled zero at the specified discount rate (target IRR). The NPV is defined as

2 $$NPV=\sum_{t=0}^{N}\frac{{CF}_{t}}{{\left(1+r\right)}^{t}}$$

where CF_t_ is the net cash flow in year *t*, *r* is the discount rate (IRR), and *n* is the project lifetime, assumed to be 15 years in this study.

The MPSP corresponds to the product selling price that satisfies

3 NPV=0

In SuperPro Designer, annual product revenue is calculated as

4 $$revenue={ṁ}_{product}\times MPSP$$

where *ṁ*_productis_ the annual mass flow rate of the product (kg·year^–1^). The MPSP, therefore, represents the minimum selling price required to ensure economic feasibility under the specified financial assumptions.

A total of 34 input process variables were considered during the simulations and are fully listed in Table S3. These variables include individual raw material costs, waste treatment costs for each identified emission stream (aqueous, gaseous, and solid), labor costs, financial parameters (inflation, income tax, loan interest rate, and loan period), and annual operating time. Table S3 also reports the corresponding annual consumption, annual cost, and percentage contribution to the preliminary cost structure of the API production process.^36,37^

The analysis focused on the functional unit of one kilogram of API as the primary product, with both annual CAPEX and OPEX estimated by using the SuperPro Designer economic tool.

The preliminary CAPEX was calculated at USD 7,066,000 (Table S4). The preliminary OPEX was determined and is summarized in Table 2. The results indicate that the main contributor to the annual OPEX (USD 5,776,000) is raw materials (37.11%), which represent the total cost of all bulk materials (pure components and stock mixtures) and discrete entities used as process inputs.

**Table 2:** Preliminary Data from the Annual OPEX of the API Production (Baseline)

**cost item**	**USD/year**	**USD/kg APIs**	**%**
raw materials	2,143,000	64.93	37.11
facility-dependent	1,325,000	40.15	22.94
labor-dependent	1,238,000	37.51	21.43
laboratory/QC/QA	186,000	5.63	3.21
waste treatment/disposal	884,000	26.78	15.31
total (OPEX)	5,776,000	175.00	100.00

The second-largest contribution corresponds to facility-dependent costs (22.94%), which include expenses associated with the use of the production facility. In greenfield designs, where no prior operational data are available, these costs are typically estimated as the sum of equipment maintenance, depreciation of fixed capital, and miscellaneous expenses, such as insurance, local property taxes, and factory overhead. In this study, facility-dependent costs include maintenance (6% of direct fixed capital, DFC), insurance (1% of DFC), local taxes (2% of DFC), and factory overhead (5% of DFC) in addition to depreciation.

Labor-dependent costs represent 21.43% of annual OPEX. This category includes all labor-related operating costs, excluding laboratory analyses, quality control (QC), and quality assurance (QA), which are accounted for separately under laboratory/QC/QA costs. In SuperPro Designer, labor-dependent costs are calculated at the section level. Specifically, the total labor cost (TLC) for each section is computed as the sum of the costs for the labor categories (operators, supervisors, and administrators).

Waste treatment and disposal costs account for 15.30% of annual OPEX. This category includes expenses related to the treatment or disposal of process output streams classified as wastes, such as undesirable byproducts, spent solvents, and emissions. Waste streams are categorized as being solid, aqueous, organic, or gaseous.

Laboratory/QC/QA costs contribute 3.21% to the annual OPEX. These costs cover off-line analytical testing, quality control, and quality assurance activities required to ensure compliance with product specifications, including chemical analysis and physical property characterization from raw materials to the final product.

Table S5 presents a stage-wise breakdown of these costs across the main processing steps: (i) product synthesis, (ii) isolation and purification, (iii) final purification, and (iv) crystallization and drying.

A standardized techno-economic assessment (TEA) was conducted using SuperPro Designer to integrate material and energy balance calculations. The software’s economic analysis tool provided capital and operating cost estimates, while data set generation was automated by linking SuperPro Designer with a VBA-based Excel calculator via the Microsoft Component Object Model (COM) interface. This automation enabled systematic manipulation of process variables, efficient simulation execution, and automated extraction of economic outputs.^37,38^

A total of 5,000 simulations were performed independently, varying the 34 input process variables. For each simulation run, input variables were randomly sampled by using a uniform probability distribution within the predefined lower and upper bounds reported in Table 3.

**Table 3:** Input Process Variables Considered in the API Production Process

	**parameter**	**unit**	**baseline**	**bounds**
	**raw material variables**			
1	quinaldine cost	USD/kg	32.0	(16.0–48.0)
2	isopropanol cost	USD/kg	1.1	(0.1–1.5)
3	nitrogen cost	USD/kg	1.0	(0.05–1.5)
4	sodium carbonate cost	USD/kg	6.5	(3.0–13.0)
5	hydroquinone cost	USD/kg	4.0	(2.0–10.0)
6	carbon tetrachloride cost	USD/kg	0.8	(0.2–2.0)
7	chlorine cost	USD/kg	3.0	(2.0–4.0)
8	sodium hydroxide cost	USD/kg	2.0	(0.5–5.0)
9	water for the process	USD/kg	0.1	(0.01–1.0)
10	wash water cost	USD/kg	0.1	(0.01–1.0)
	**waste cost variables**			
11	carbon tetrachloride waste cost	USD/kg	5.0	(4.0–6.0)
12	carbon dioxide waste cost	USD/kg	5.0	(4.0–6.0)
13	isopropanol waste cost	USD/kg	2.0	(1.0–3.0)
14	sodium hydroxide waste cost	USD/kg	2.0	(1.0–3.0)
15	charcoal waste cost	USD/kg	2.0	(1.0–3.0)
16	quinaldine waste cost	USD/kg	2.0	(1.0–3.0)
17	chloroquinaldine waste cost	USD/kg	2.0	(1.0–3.0)
18	product waste cost	USD/kg	2.0	(1.0–3.0)
19	product crystal waste cost	USD/kg	2.0	(1.0–3.0)
20	impurity waste cost	USD/kg	2.0	(1.0–3.0)
21	product.Na waste cost	USD/kg	2.0	(1.0–3.0)
22	chlorine waste cost	USD/kg	2.0	(1.0–3.0)
23	hydrochloric acid waste cost	USD/kg	2.0	(1.0–3.0)
24	hydroquinone Na waste cost	USD/kg	2.0	(1.0–3.0)
25	methanol waste cost	USD/kg	2.0	(1.0–3.0)
26	sodium carbonate waste cost	USD/kg	2.0	(1.0–3.0)
27	hydroquinone waste cost	USD/kg	2.0	(1.0–3.0)
28	sodium chloride waste cost	USD/kg	2.0	(1.0–3.0)
	**labor variables**			
29	labor cost	USD/hour	41.4	(5.0–50.0)
	**financial variables**			
30	inflation	%	4	(1.0–6.0)
31	income taxes	%	40	(10.0–50.0)
32	loan interest	%	9	(1.0–10.0)
33	loan period	years	10	(3.0–12.0)
34	annual operation time	hours	7,920.00	(7,200.0–8,760.0)

The lower and upper bounds assigned to each variable were defined based on market price fluctuations reported in industrial databases and literature, historical commodity price variability (±25–50% for major raw materials), the SuperPro Designer economic database for waste treatment costs, and financial scenario ranges commonly adopted in techno-economic studies.

For raw material costs, the selected bounds reflect realistic market volatility observed for bulk chemicals used in pharmaceutical manufacturing.^39^ Waste treatment cost ranges were established on the basis of typical disposal fees for aqueous, gaseous, and hazardous organic waste streams in industrial practice.^40^

Financial variables were bounded according to equity values and the degree of investment by investors for a sample of chemical and drug development companies: inflation (1–6%), representing low to high macroeconomic scenarios;^41–43^ income tax bounds were set between 10 and 50% (0.10 to 0.50 in decimal format, as shown in Figure 6h). This interval reflects the global disparity between nominal and effective corporate tax rates and encompasses a broad range of potential international taxation scenarios. The loan interest rate (1–10%), covering low- to high-risk financing conditions; loan period (3–12 years), representing short- to long-term financing structures; and annual operating time (7,200–8,760 h year^–1^), corresponding to 300 operating days up to continuous 365-day operation.

These intervals were selected to represent realistic yet conservative uncertainty ranges, enabling robust stochastic techno-economic assessment while avoiding infeasible process conditions.

The primary raw material variables included quinaldine, hydroquinone, carbon tetrachloride, chlorine, isopropanol, sodium hydroxide, sodium carbonate, nitrogen, and water. Labor costs were represented by a single category (“Operator”) with a variable hourly wage. Administrative and supervisory costs were modeled as functions of the labor rate, if higher wages for skilled professionals correspond to lower relative administrative burdens, and vice versa. For labor rates below USD 5 h^–1^, the administration factor was set to 0.9; for USD 5–10 h^–1^, 0.5; for USD 10–15 h^–1^, 0.3; and for rates above USD 18 h^–1^, 0.1. Similarly, the supervision factor ranged from 0.8 at the lowest wages to 0.2 at the highest wages. These adjustments ensured proportional alignment among labor, administrative, and supervisory costs, remaining consistent with the U.S. federal minimum wage of USD 7.25 h^–1^.^44^

Waste treatment costs were obtained directly from the SuperPro economic database. Financial variables affecting the facility-dependent cost included inflation, income tax, loan interest rate, and loan period. Annual operating time was evaluated within two bounds: (i) 7,200 h year^–1^ (300 operating days), accounting for maintenance and administrative downtime, and (ii) 8,760 h year^–1^ (continuous operation), representing a theoretical no-stoppage scenario.

Random numbers were generated in VBA using the Rnd­() function, which produces pseudorandom values between 0 and 1. Each variable was sampled according to

5 $$X_{i}=X_{\min}+\mathrm{Rnd}\left(\right)\times \left(X_{\max}-X_{\min}\right)$$

where *X_i_* is the sampled value, and *X*_min_ and *X*_max_ correspond to the lower and upper bounds, respectively.

A uniform distribution was adopted for all variables because insufficient statistical data were available to justify parametric distributions. Furthermore, this approach ensures an unbiased exploration of the entire feasible parameter space across the defined uncertainty ranges.

Each sampled input set was transmitted automatically to SuperPro Designer via the COM interface, where mass balances, energy balances, and economic calculations were executed.

For each simulation run, the 34 independent variables were randomly sampled, while UPC, MPSP, and IRR were computed as economic outputs. The resulting MPSP, UPC, and IRR were exported back to Excel and compiled into the final data set.

### 2.3 ML Model Tuning

Effective predictive modeling in techno-economic analysis requires a well-structured data set and optimized ML models. In this study, data generation was critical to ensuring representative system conditions, enabling robust predictions of economic and operational metrics. This section outlines the workflow for data set construction and model fine-tuning, as illustrated in Figure 2, detailing how process simulations were used to create training data and how ML techniques were applied to identify key patterns, make predictions, and enhance model performance.

**Figure 2:**
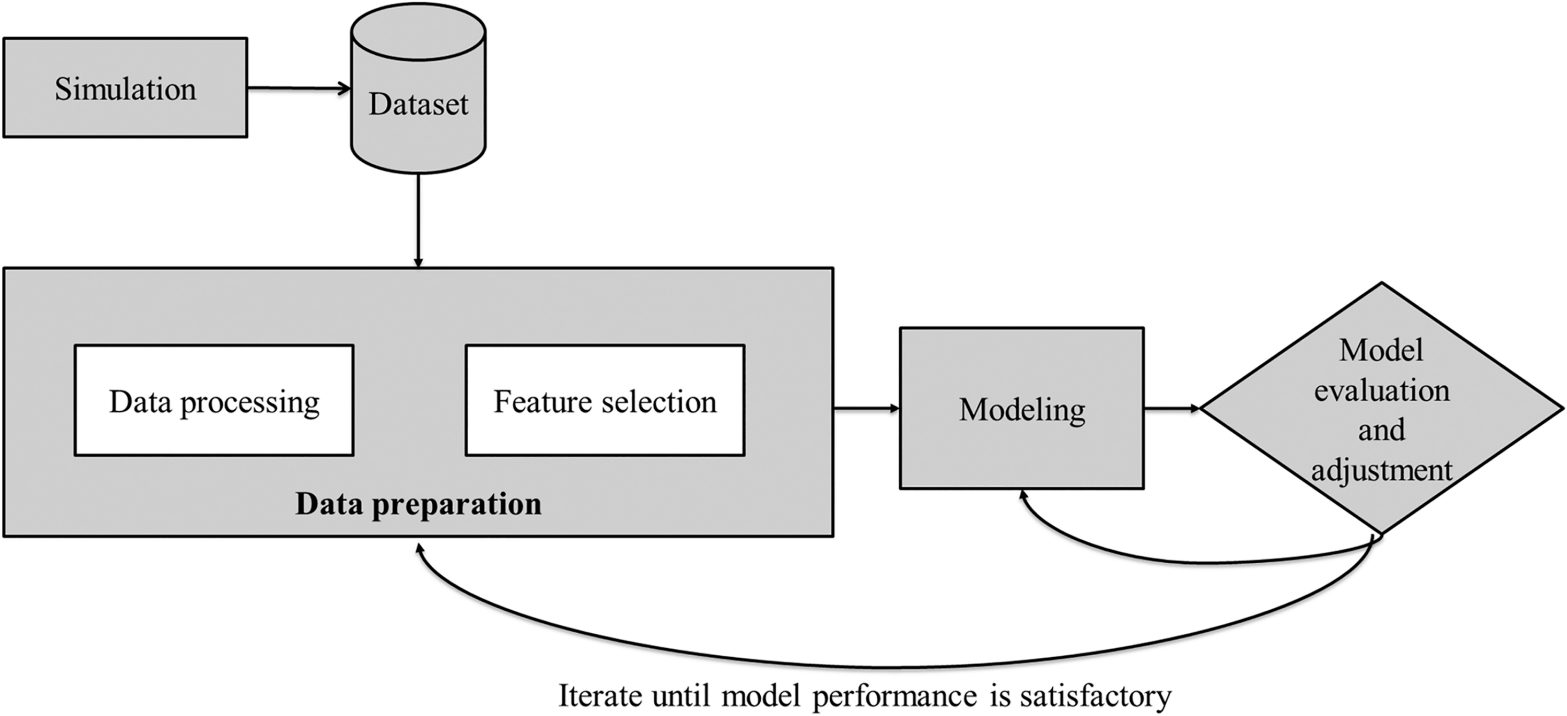
Model development process flow.

Given the large number of variables in the generated data set, a Pearson correlation matrix and the least absolute shrinkage and selection operator (LASSO) regression were applied to identify those most strongly associated with the target variables. The final data set comprises 37 variables, but only two of them are the ML targets: UPC and MPSP. Although the IRR is an output variable derived from the techno-economic evaluation, in this study, it was intentionally treated as an input feature in the machine learning model. This modeling choice reflects a goal-oriented design perspective in which the user of the model specifies a target IRR as a desired economic performance criterion.

LASSO is a regression method that performs variable selection and regularization by shrinking the coefficients of less relevant predictors toward zero, thereby reducing overfitting.^45^ To maximize model efficiency, the multitask LASSO algorithm was employed to jointly model MPSP and UPC, enabling the simultaneous identification of features with predictive relevance for both target variables. This multivariate approach leverages potential correlations between the targets, improving the feature selection consistency and reducing model complexity.

The correlation matrix displays Pearson coefficients, which quantify the strength and direction of linear relationships between variables.^46^ The Pearson correlation coefficient (r) ranges from −1 (strong negative correlation) to 1 (strong positive correlation), with 0 indicating no linear relationship.^47^ This matrix is widely used in exploratory data analysis and feature selection to identify highly correlated predictors and minimize multicollinearity in modeling tasks.^48^ This approach enhances predictive performance while reducing data set dimensionality.

Several ML models were tested for predicting key indicators. Model selection was based on performance metrics, including the *R* ^2^, RMSE, and MAPE, as defined by eqs 6 to 8, where *y*_*i*_ represents the original data points and *ŷ*_*i*_ corresponds to the model predictions. The RMSE penalizes larger errors, making it sensitive to outliers and useful for assessing overall accuracy.^49^ The MAPE evaluates errors in percentage terms, facilitating performance interpretation relative to actual values,^50^ while *R* ^2^ measures the proportion of variability explained by the model, essential for assessing predictive capacity.^51^

6 $$R^{2}=1-\frac{\sum_{i=1}^{n}{\left(y_{i}-{\hat{y}}_{i}\right)}^{2}}{\sum_{i=1}^{n}{\left(y_{i}-\mathrm{y̅}\right)}^{2}}$$

7 $$\mathrm{RMSE}=\sqrt{\frac{1}{n}\sum_{i=1}^{n}{\left(y_{i}-{\hat{y}}_{i}\right)}^{2}}$$

8 $$\mathrm{MAPE}=\frac{1}{n}\sum_{i=1}^{n}\left|\frac{y_{i}-{\hat{y}}_{i}}{y_{i}}\right|\times 100$$

Six ML techniques were evaluated: linear regression (LR), k-nearest neighbors’ regression (KNN), random forest, polynomial ridge, support vector regression (SVR), and extreme gradient boosting (XGBoost). LR served as a baseline due to its simplicity and interpretability. However, its limitations, such as sensitivity to outliers and inability to capture complex relationships, led us to explore more advanced models.^52^ KNN, a nonparametric method, captures nonlinear patterns, but it is computationally intensive for large data sets.^53^ Random forest reduces overfitting through ensemble learning,^54^ while polynomial ridge regression handles nonlinear relationships with ridge regularization.^55^ SVR uses kernel functions for robust performance in linear and nonlinear scenarios,^56^ and XGBoost iteratively combines weak decision trees to minimize errors.^57^

The data set was initially partitioned into 80% for training and 20% for testing using the hold-out method. Within the training set, model selection was carried out using 5-fold cross-validation, ensuring a robust internal performance estimation and preventing information leakage. The test set was reserved exclusively for the final performance assessment of the selected optimal models. This approach is well established in the literature as providing lower variance in performance estimation and is recommended for model selection and validation in real-world scenarios.^58^

For the initial performance analysis, all models were trained using their default hyperparameters and evaluated using the full set of input features of the train set (35 variables as aforementioned). In the second stage, all models were retrained, still using default hyperparameters, but using only the subset of features selected through the feature selection process. For the best-performing model identified, hyperparameter optimization was performed using randomized search cross validation to determine the optimal parameter configuration. In contrast to grid search, which exhaustively evaluates all possible parameter combinations, the technique of randomized search samples hyperparameter values from predefined distributions. This approach achieves comparable performance to the grid search technique while dramatically reducing computational overhead, particularly in high-dimensional search spaces.^59^ As the final step, the model’s generalization performance was evaluated on the hold-out test set.

The process was conducted within a multioutput regressor, enabling the simultaneous prediction of MPSP and UPC, which treats each target as an independent regression problem. For each model, evaluation metrics were calculated independently for each target variable and then averaged using the multioutput = ’uniform_average’ parameter in Scikit-learn. Figure 3 illustrates the workflow employed for the determination of the optimal model.

**Figure 3:**
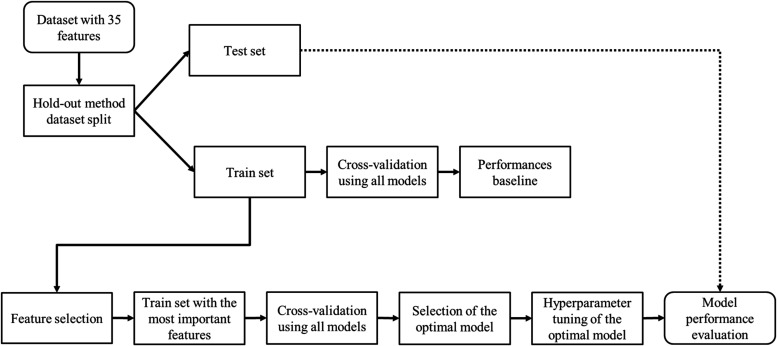
Workflow for optimal model determination.

To ensure that all variables contribute equally to the model and to prevent features with larger numerical ranges from dominating the learning process, normalization was performed using min–max scaling. In each fold of the cross-validation, the scaling parameters (minimum and maximum values) were computed exclusively from the training subset and subsequently applied to both the training and validation subsets, rescaling all features to the [0, 1] range, as defined in eq 9. This preprocessing step is especially important for algorithms sensitive to feature magnitude. Recent studies have demonstrated that normalization methods, if not carefully applied, can significantly influence model behavior and confound downstream performance, particularly in complex and heterogeneous data sets.^60^

9 $$x_{\mathrm{scaled}}=\frac{x-x_{\min}}{x_{\max}-x_{\min}}$$

To enable a fair and interpretable comparison between the model’s predictive error and the inherent variability of each target variable, min-max normalization was applied to the output data (MPSP and UPC) in the final stage of the workflow. This normalization was performed only for the best-performing model, previously optimized via randomized search, and was not part of the training procedure for earlier models. The two target variables operate on different scales and orders of magnitude and rescaling them to a common [0, 1] range makes it become possible to interpret the RMSE in a normalized context. This allowed for a meaningful comparison of model error relative to the total variation in the data, providing a more robust evaluation of predictive performance across targets.

All models were implemented using Google Colaboratory, which provides GPU/TPU support for efficient computation and seamless integration with Python libraries. Uncertainty analysis was conducted using kernel density estimation (KDE) to visualize variable distributions and Shapley additive explanations (SHAP) to interpret feature importance and quantify their impact on model predictions.^61,62^ These techniques provided a comprehensive understanding of how input variations influence the MPSP and UPC, emphasizing the importance of uncertainty quantification in predictive modeling.

To analyze the relationship among the MPSP, IRR, and UPC, a two-dimensional interpolation technique was employed. Specifically, a cubic interpolation method was used to generate a heatmap, allowing for a smooth and continuous representation of revenue variations under different economic conditions. This approach improves visualization by effectively capturing the intricate interactions between investment returns and production costs while mitigating the influence of minor data fluctuations.^63,64^

Following the principle of parsimony, the analyses commenced with simpler models and progressively incorporated more complex and robust approaches.This methodological progression ensured a balance between interpretability and predictive power, allowing for a comprehensive evaluation of the model performance.

## 3 Results and Discussion

This section presents the application of ML models to predict key economic indicators associated with the batch production of the API synthesized via the condensation of quinaldine and hydroquinone. An uncertainty analysis is subsequently conducted, followed by a discussion of profitability-related parameters. First, ML-based estimators for the MPSP and UPC are developed, integrating 34 cost-related parameters and the IRR. The models offer a data-driven framework for assessing the economic feasibility of the processes.

Next, an uncertainty analysis is conducted by KDE and SHAP to quantify variability and identify the key cost drivers. Finally, a discussion explores the relationship among MPSP, IRR, and UPC using a two-dimensional interpolation heatmap, visualizing revenue distribution under different economic conditions. This analysis highlights the sensitivity of profitability to fluctuations in production costs and investment returns, providing insights into the optimization of financial performance.

### 3.1 Machine Learning-Based Estimators for MPSP and UPC

The proposed framework first defines the distributions and bounds of 34 input variables, along with the IRR. The IRR directly influences the MPSP by determining the price required to achieve a target return on investment; higher IRR requirements lead to an increased MPSP. Additionally, the UPC impacts this relationship as a higher UPC requires a higher MPSP to sustain profitability. Together, IRR, MPSP, and UPC define the economic feasibility of the process, ensuring that revenue covers production costs while meeting return expectations. Using well-trained ML models, a multivariate stochastic TEA is conducted, where these variables are randomized simultaneously to assess their combined impact on the two target outputs: the UPC and the MPSP per kilogram of API produced. This approach enables a comprehensive assessment of economic feasibility. Table 4 presents the performance of the ML models based on evaluation metrics, considering all 35 input variables and their influence on UPC and MPSP, establishing a baseline for comparison.

**Table 4:** Average Performance on the Validation Folds Obtained through Cross-validation Using the Training Set with All Available Features

**models**	***R*** ^**2**^	**RMSE**	**MAPE (%)**
KNN	0.29	174.41	29.31
SVR	0.45	171.93	28.56
linear regression	0.60	155.43	22.03
polynomial ridge	0.74	125.45	16.66
random forest	0.84	**88.28**	10.48
XGBoost	**0.86**	88.70	**9.88**

The initial evaluation, in which all models were trained with default hyperparameters and the complete set of input variables, revealed significant differences in the predictive performance. Linear regression, SVR, and polynomial ridge models exhibited moderate predictive capabilities, with R^2^ values between 0.45 and 0.74. The poor performance of KNN (*R* ^2^ = 0.29) suggests its limitations in handling high-dimensional data.

In contrast, XGBoost demonstrated optimal predictive performance, achieving the highest *R* ^2^ (0.86) and the smallest MAPE (9.88%), indicating a strong fit to the original data. Both random forest and XGBoost achieved similarly high predictive performance on the training set, with random forest yielding the lowest RMSE value (88.28). This similarity can be attributed to the fact that both algorithms are ensemble methods based on decision trees, which allow them to model complex nonlinear relationships and capture high-order feature interactions. Random Forest combines the predictions of multiple decorrelated decision trees through bagging, reducing variance, and improving generalization. XGBoost, on the other hand, builds trees sequentially using gradient boosting, optimizing the residual errors from previous iterations, and incorporating regularization to control overfitting. This methodological framework enabled the algorithm to achieve marginally superior results in most of the evaluated metrics, thereby establishing it as the top-performing model in this assessment.

While these initial results provide valuable insights, not all variables contribute equally to the model’s predictive performance. An exploratory analysis using Pearson’s correlation revealed no strong linear relationships between input variables and target metrics (UPC and MPSP), as shown in the correlation matrix in Figure S1. The absence of significant correlations (most *r* < 0.5) between predictors and targets fundamentally constrained the model’s ability to establish meaningful patterns during fitting. While some variables exhibited complex interdependencies, the default correlation analysis failed to identify clear feature selection criteria, prompting the need for alternative approaches.

To address these challenges, multitask LASSO regression was applied to identify and retain the most informative predictors. This approach refined the models by eliminating less-relevant variables, enhancing overall predictive performance, and improving interpretability. The analysis identified a set of 9 variables as having a significant impact on the MPSP and UPC targets, with the corresponding feature importance scores reported in Table S6. These include key cost-related factors, such as labor cost and the prices of critical raw materials (quinaldine, isopropanol, water, and nitrogen); operational parameters, such as the annual operation time; and financial indicators, such as the income tax rate, loan period, and IRR. These drivers highlight the combined influence of financial planning, operational efficiency, raw material management, and workforce expenses on cost estimation and revenue prediction. Importantly, these variables not only affect the UPC but also directly impact the MPSP, which in turn influences the IRR. Understanding these interdependencies enables the model to better capture the economic dynamics of API production, positioning the MPSP as a central metric for profitability assessment and decision-making.

After identifying the 9 most relevant features, all models were retrained using the reduced feature train set while maintaining default hyperparameter configurations. This step aimed to eliminate noise and redundancy, enabling the models to focus on the most influential predictors and improve generalization as well as computational efficiency. Table 5 presents the updated models’ performances after the feature selection procedure.

**Table 5:** Average Performance on the Validation Folds Obtained through Cross-validation Using the Reduced Training Set after Feature Selection

**models**	***R*** ^**2**^	**RMSE**	**MAPE (%)**
SVR	0.53	163.52	26.04
linear regression	0.59	155.05	22.40
KNN	0.64	115.19	17.83
polynomial ridge	0.78	112.55	15.14
random forest	**0.85**	90.53	9.88
XGBoost	**0.85**	**87.47**	**9.74**

The results indicate that feature selection led to significant performance improvements across most models, particularly KNN, random forest, and polynomial ridge. The reduction in dimensionality helped to minimize overfitting and computational complexity, allowing models to generalize better to unseen data.

Notably, KNN experienced the most substantial improvement, with *R* ^2^ increasing from 0.29 to 0.64 and a reduction in RMSE and MAPE. This suggests that removing features with less relevance to the target variables enhanced the model’s ability to capture meaningful patterns. SVR and polynomial ridge also showed significant gains in predictive performance, reinforcing the effectiveness of feature selection in reducing noise and improving model stability.

Despite a slight reduction of 0.01 in *R* ^2^, both XGBoost and random forest reached a value of 0.85. However, XGBoost delivered the best overall performance, attaining the lowest RMSE (87.47) and the lowest MAPE (9.74%), thereby establishing it as the top-performing model in this evaluation. While predictive accuracy remained largely unchanged, feature reduction led to substantially lower computational cost and faster training times, with training time decreasing from 4.42 to 2.79 s for XGBoost and from 1 min 3 s to 16.4 s for Random Forest, indicating improved model efficiency.

To complement the aggregated evaluation, separate analyses of UPC and MPSP are conducted. Although both targets are closely related economic indicators, they represent distinct cost metrics within the techno-economic framework and are derived through different computational procedures. As a result, differences in the model behavior and predictive performance between these two targets are expected. To better understand these differences and clarify the relationships between the target variables and the input features, correlation analysis is presented.

Building on these target-specific insights, complementary quantitative and visual assessments were performed to capture both overall accuracy and prediction dispersion. For UPC prediction, all models yielded strong predictive performance, with consistently high *R* ^2^ values (≥0.92) and low MAPE values (≤4%), as detailed in Figure S2. This indicates that, regardless of the regression technique applied, the models were able to capture the underlying behavior of the UPC variable with minimal relative error. In contrast, the scatter plots of predicted versus actual MPSP values shown in Figure 4 reveal a more challenging prediction task, with greater dispersion around the ideal regression line, highlighting the increased complexity associated with MPSP estimation.

**Figure 4:**
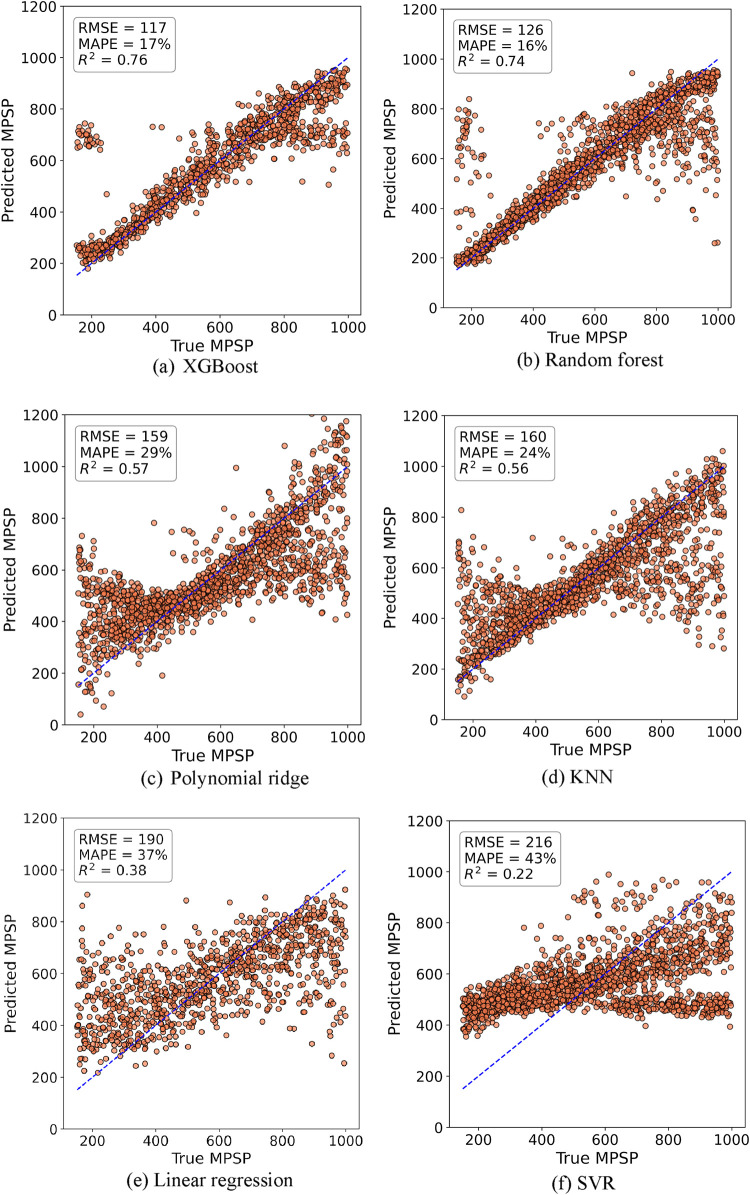
Predicted vs actual values for MPSP across different ML models after the execution of the feature selection: (a) XGBoost, (b) Random forest, (c) Polynomial ridge, (d) KNN, (e) Linear regression, and (f) SVR.

To further interpret these results, a correlation analysis based on Spearman’s rank correlation coefficient (eq 9) was conducted to investigate the relationships between the target variables and the input features. This analysis allows the identification of monotonic associations without assuming linearity or normal data distributions, where correlation coefficients closer to 1 indicate stronger positive monotonic relationships between variables.

10 $$\rho =1-\frac{6\sum d_{i}^{2}}{n\left(n^{2}-1\right)}$$

The results indicate that the variables strongly associated with each target are substantially different. UPC is primarily influenced by direct operational cost variables, which enter its calculation in a relatively straightforward manner, facilitating accurate learning by regression models. In contrast, MPSP only exhibits a considerable dependence on IRR. Unlike UPC, MPSP is not obtained through a direct analytical expression but is determined iteratively by adjusting the product selling price until a target internal rate of return is achieved. This indirect and nonlinear calculation increases sensitivity to multiple financial parameters, providing a rationale for the comparatively lower predictive performance observed for the MPSP. The correlation results are summarized in Table 6.

**Table 6:** Spearman Correlation Coefficients between Input Features and Target Variables

**UPC**		**MPSP**	
**feature**	**Spearman correlation**	**feature**	**Spearman correlation**
labor (USD/h)	0.73	IRR (%)	0.38
quinaldine (USD/kg)	0.50	methanol waste (USD/kg)	0.03
isopropanol (USD/kg)	0.30	water (USD/kg)	0.03
water (USD/kg)	0.25	carbon dioxide waste (USD/kg)	0.02
nitrogen (USD/kg)	0.13	product waste (USD/kg)	0.02

Notably, for the MPSP target, XGBoost remained the best-performing model, showing the closest alignment to the diagonal reference line and reaffirming its superior predictive capability.

Given that the XGBoost model demonstrated the optimal performance, a randomized search cross-validation strategy was employed to optimize its hyperparameters. The hyperparameter space explored during this process is summarized in Table 7. The selected parameter ranges were informed by relevant literature and preliminary testing, ensuring a balanced and thorough exploration of configurations to reduce the risk of both overfitting and underfitting. Optimization was carried out using 5-fold cross-validation over 30 iterations on the reduced training set, providing a robust assessment of model performance across different data splits.

**Table 7:** Hyperparameter Space Explored During the XGBoost Optimization Process Using Randomized Cross-Validation Search

**hyperparameters**	**description**	**range**
number of estimators	number of trees in the ensemble	50–300
maximum depth (max depth)	controls the maximum depth of each decision tree	3–30
learning rate	shrinks the contribution of each tree to prevent overfitting	0.001–0.1
subsample	fraction of training instances randomly selected to train each tree	0.3–1.0
column sample by tree	fraction of features (columns) randomly sampled for each tree	0.3–1.0

Building on this tuning process, the results obtained after feature selection are shown next, allowing for a direct comparison with the full-variable approach presented earlier.

The best-performing configuration for the XGBoost-based multioutput regression model included a subsample ratio of 0.8, 173 estimators, a maximum tree depth of 3, a learning rate of 0.1, and a column sample by tree value of 1.0. The model achieved an average *R* ^2^ score of 0.86 in the validation set. The best hyperparameters found in this process were then applied to estimate additional metrics on the reduced training set, resulting in an average RMSE of 87.84 and an MAPE of 9.71% in the validation set. Although the results are very close to those obtained with the initial default configuration, hyperparameter tuning slightly outperformed the baseline in terms of *R* ^2^ and MAPE, indicating a modest but consistent gain. One possible explanation is that the default hyperparameters were already well-calibrated for the data distribution and problem complexity, positioning the model near its optimal performance.

Additionally, Figure 5 displays the learning curves for the XGBoost model trained with the selected 9 variables and the best hyperparameters, illustrating the evolution of training and validation *R* ^2^ scores as the training set increases. The validation curve shows steady improvement, suggesting that the model generalizes well with more data. Furthermore, the narrowing gap between training and validation scores demonstrates XGBoost’s ability to balance bias and variance effectively.

**Figure 5:**
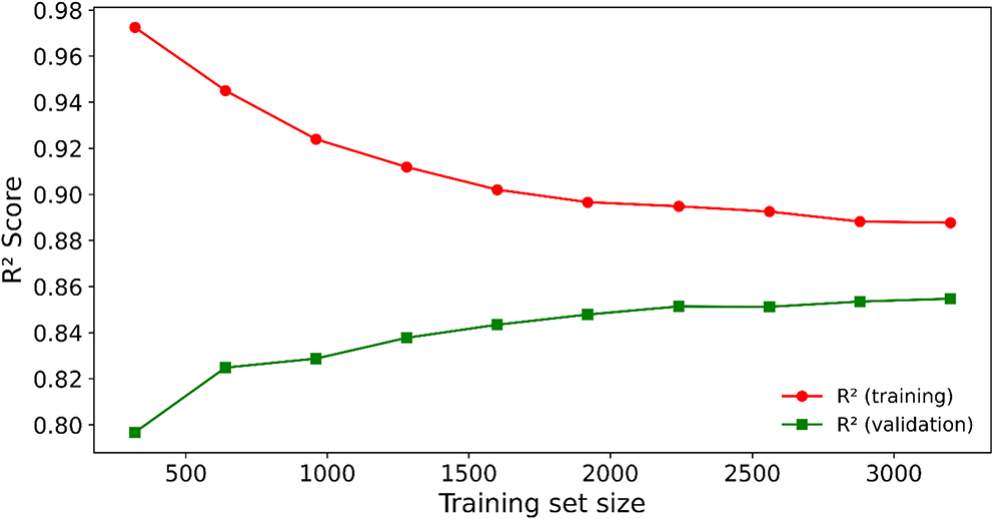
Learning curves for the best XGBoost model trained with the selected features.

The model’s performance was evaluated on the hold-out test set to assess its generalization capability. The model achieved an *R* ^2^ of 0.87, an RMSE of 85.21, and a MAPE of 9.36% on the test data. These results indicate that the performance was preserved, confirming the model’s ability to generalize beyond the training data without significant overfitting.

After hyperparameter optimization via randomized search, the XGBoost model was retrained using min-max normalized target variables (MPSP and UPC) to enable a more interpretable assessment of predictive performance relative to the inherent variability of each output. This normalization step, applied only at this final evaluation stage, allowed the RMSE values to be meaningfully compared against the normalized scale of the targets. The results demonstrated strong model performance: for MPSP, the RMSE was 0.1442, approximately half the standard deviation of the observed normalized values (0.2926), indicating that the model accounts for a substantial portion of the output’s variability. For UPC, the RMSE was 0.0256, equivalent to about 15% of the normalized standard deviation (0.1741), which reflects very low predictive error relative to the variation in the data. These findings confirm that the optimized XGBoost model effectively captures the behavior of both target variables, especially in the case of UPC, where the prediction error is minimal compared to the scale of the response.

These findings highlight the advantages of integrating LASSO-based feature selection into the modeling pipeline. While the IRR and 34 input process variables (with boundaries detailed in Table 3) were initially considered, LASSO’s constrained optimization identified just 9 variables (26% of original features) that substantially affect both MPSP and UPC predictions. This efficient feature selection challenges conventional assumptions about key economic drivers, reduces computational overhead, and improves model generalizability, which are three critical advantages for achieving a robust techno-economic analysis.

The results following feature selection highlight the benefits of reducing dimensionality to improve model performance. By retaining only the nine most relevant variables: labor costs, raw material costs (quinaldine, isopropanol, water, and nitrogen), financial and operation factors (income taxes, loan period, and annual operation time), and IRR, all models achieved higher predictive accuracy, with XGBoost consistently outperforming the others. The comparative scatter plots confirm the enhanced alignment between predictions and actual values, while the learning curves underscore XGBoost’s strong generalization capability.

These findings emphasize the critical role of feature selection in refining ML models, leading to more interpretable, efficient, and accurate predictions for both the MPSP and UPC.

### 3.2 Uncertainty Analysis

Understanding the uncertainty in predictive modeling is crucial for assessing the reliability of results and identifying the factors that introduce variability into predictions. This section explores uncertainty using KDE visualization, complemented by SHAP values for feature importance. These analyses provide insights into the distribution of key variables, their impact on model outputs, and how uncertainty propagates through predictions of the MPSP and UPC.

To further assess uncertainty in financial performance, a KDE plot was utilized to illustrate the distribution of key features on MPSP and UPC. These analyses, presented in Figure 6, highlight the overall influence of critical financial and operational parameters. The KDE (blue curve) represents the probability density functions (PDFs), revealing whether variable distributions are normal, skewed, or multimodal.

**Figure 6:**
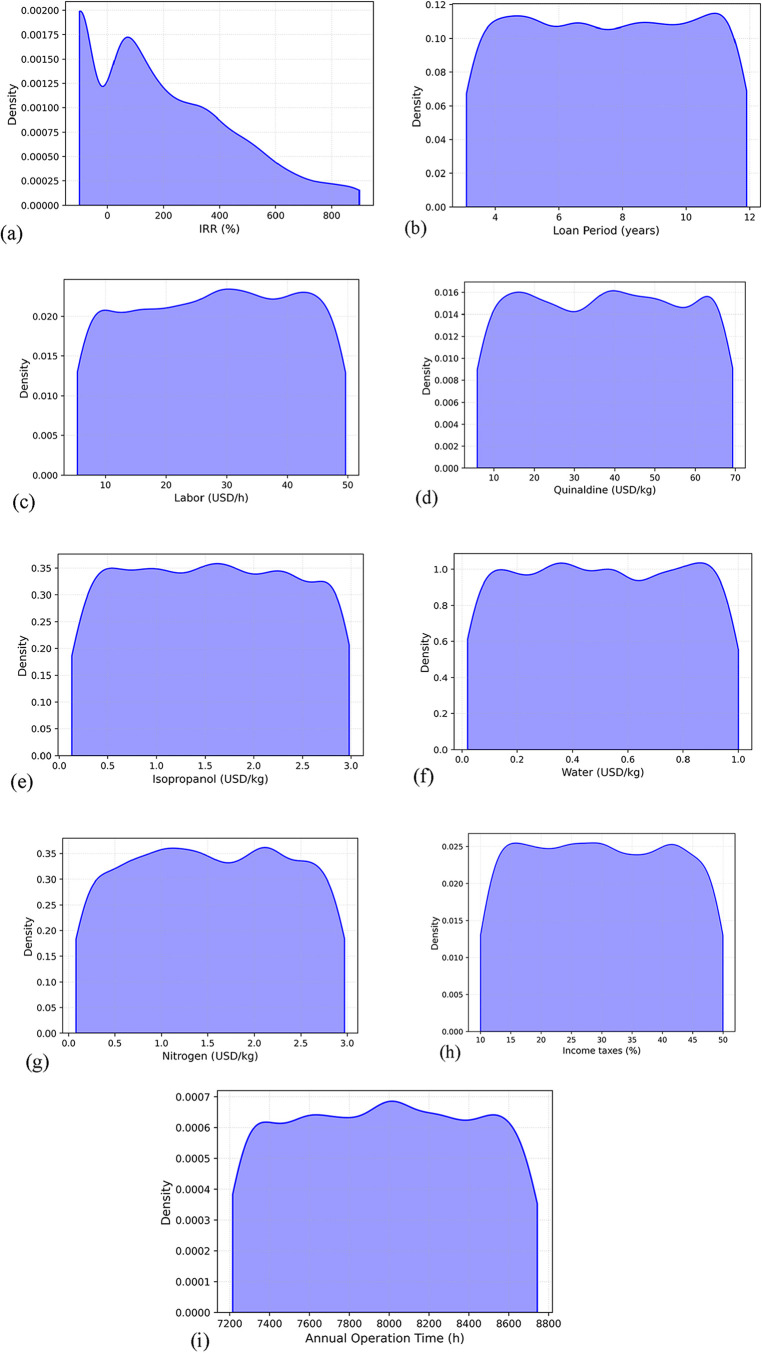
KDE plots illustrate the distribution of the most relevant features: (a) IRR (After taxes), (b) Loan period, (c) Labor, (d) Quinaldine cost, (e) Isopropanol cost, (f) Water cost, (g) Nitrogen cost, (h) Income taxes, and (i) Annual operation time.

The KDEs indicate heterogeneity in the statistical properties of financial and operational inputs. IRR (after taxes) displays a positively skewed and multimodal distribution, with a dominant peak at low-to-moderate values and a long right tail reflecting rare high-return scenarios. Loan period, labor, quinaldine cost, isopropanol cost, nitrogen cost, water cost, and income taxes exhibit approximately uniform to mildly multimodal distributions, characterized by low skewness and flat kurtosis, suggesting that uncertainty is distributed relatively evenly across their ranges. Annual operation time follows a narrow, symmetric distribution with low variance, indicative of operational constraints limiting variability. Collectively, these distribution profiles reveal that variables with higher skewness and kurtosis (e.g., IRR) have greater potential to propagate extreme values into MPSP and UPC predictions, while flatter or symmetric distributions contribute more consistent effects.

To further assess uncertainty, SHAP values were computed to quantify the contribution of each feature to model predictions, as shown in Figure 7. SHAP values enable the interpretation of how individual variables influence MPSP and UPC under varying conditions. The summary plot shows both how important each feature is and how it affects the model’s predictions. Each point represents a Shapley value for one feature in a specific data point. Features are listed on the *y*-axis, while the *x*-axis shows whether that feature pushed the prediction higher or lower. The color of the points reflects the feature’s actual value, from low to high, helping to visualize how different ranges of the feature influence the output.

**Figure 7:**
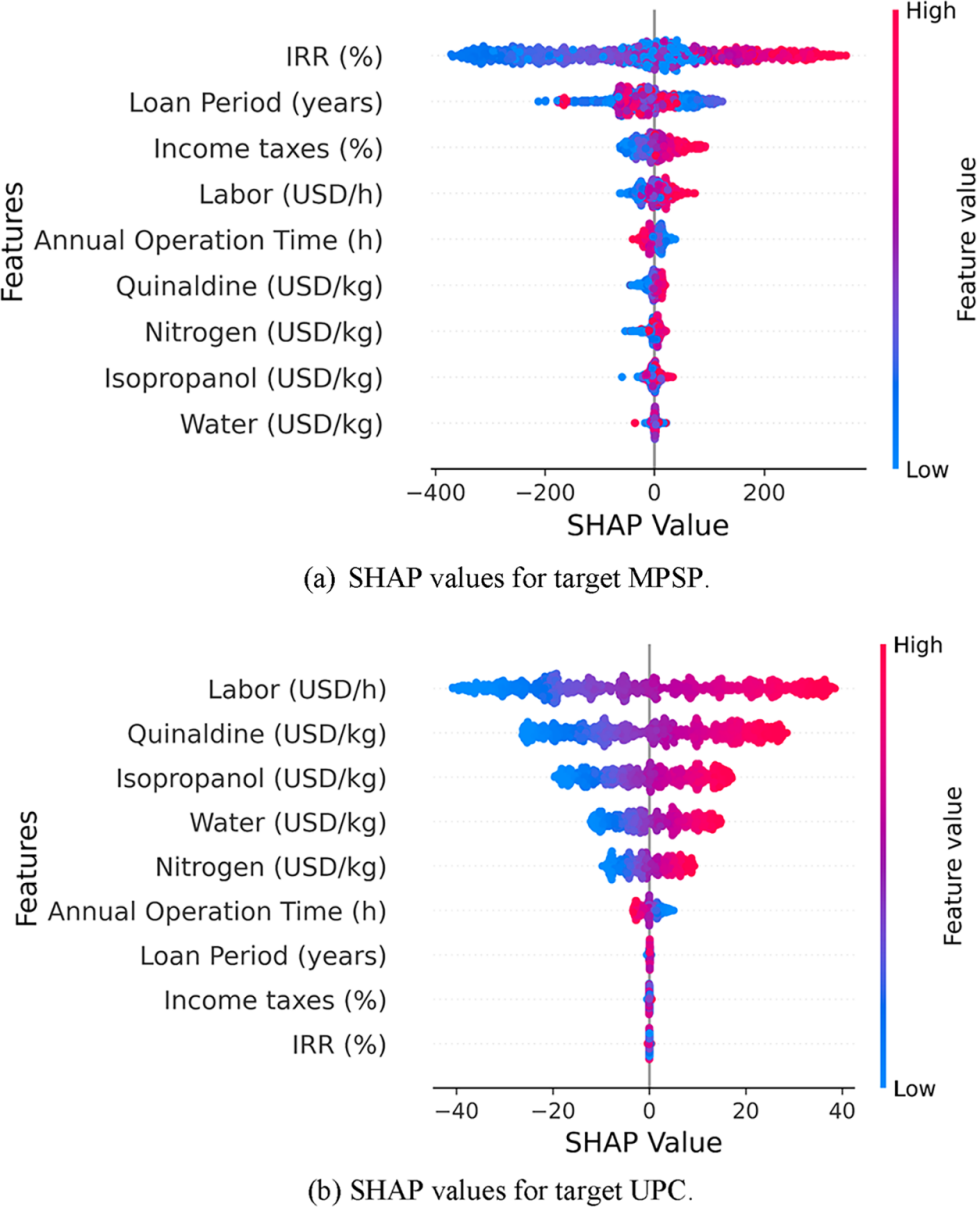
Model interpretability through SHAP summary plots for (a) target MPSP and for (b) target UPC.

The SHAP summary plot reveals that IRR (after taxes) is the most influential factor in predicting MPSP, exerting a substantial effect on revenue variability. While the loan period also contributes, its influence is notably smaller. These results emphasize the importance of investment-related variables in shaping the revenue outcomes. Extreme values of IRR and tax rates introduce considerable uncertainty into MPSP estimates, indicating a high sensitivity to both the capital investment conditions and fiscal policies. For UPC predictions, the most influential drivers are the labor costs and raw material prices. The magnitude of their SHAP values indicates that variations in operational and material expenses substantially contribute to the uncertainty in the production cost estimates.

By comparing KDE distributions with SHAP values, a distinction emerges between features that not only exert a strong influence but also contribute to uncertainty. Loan period and income taxes exhibit wide KDE distributions and high SHAP variability, demonstrating their inconsistent effects on the MPSP across different scenarios. These financial parameters introduce significant uncertainty into pricing predictions.

Conversely, water and nitrogen costs exhibit lower SHAP dispersion and more stable probability distributions, implying a more predictable influence on UPC. This suggests that fluctuations in these costs have a relatively consistent impact on the production costs.

Understanding these distinctions is crucial for strategic decision making. Features associated with high uncertainty require robust financial and operational controls to mitigate risks, whereas more stable variables serve as reliable indicators for cost estimations.

Learning curve analyses indicate that while model performance improves with additional training data, uncertainty remains high for variables with a greater SHAP variability. The IRR (after taxes) exhibits a broad SHAP value range, signifying nonuniform effects on revenue. This suggests that while the model captures overall economic trends, financial indicators fluctuate on the basis of specific market conditions.

The SHAP plots confirm that investment- and tax-related features exhibit dynamic interactions, reinforcing the necessity for continuous financial monitoring and adaptive planning.

This uncertainty assessment highlights the importance of understanding the feature distribution, impact variability, and model sensitivity. High-uncertainty features (loan period, income taxes, and IRR (after taxes)) require vigilant monitoring to ensure robust financial planning and risk management. Conversely, more stable variables (water cost, raw material prices) provide consistent predictive value, making them reliable indicators for production cost estimations. By integrating KDE visualizations with SHAP analysis, this study offers a comprehensive approach to uncertainty assessment, enabling more informed financial and operational decision making.

### 3.3 Profitability Parameters: MPSP, IRR, and UPC

To evaluate the relationship among UPC, IRR, and MPSP, a two-dimensional interpolation approach was applied using the optimal XGBoost-based multioutput regression model described in section 3.1. A heatmap (Figure 8) was generated using cubic interpolation of the model predictions, providing a smooth and continuous representation of the revenue behavior across varying economic conditions. This visualization captures the nonlinear interaction between the investment return and production cost while minimizing the influence of minor data fluctuations.

**Figure 8:**
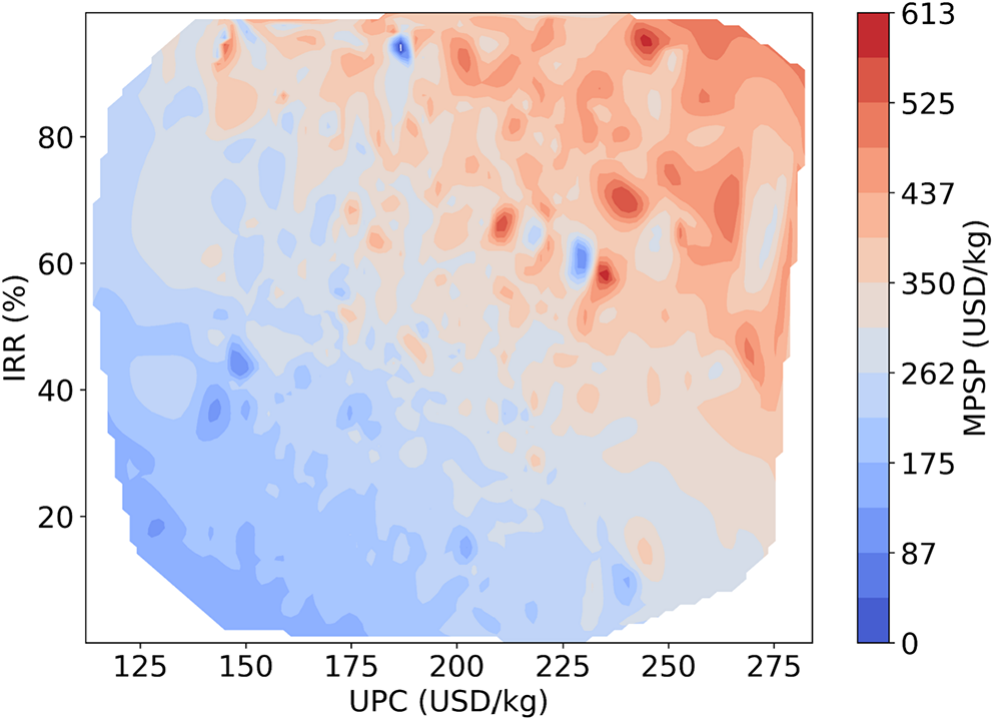
Heatmap of MPSP as a function of IRR (after taxes) and UPC: Identifying economic feasibility regions.

In the heatmap, the color gradient represents MPSP levels: warmer tones (red) correspond to higher MPSP values, whereas cooler tones (blue) indicate lower values. High-revenue regions are predominantly located in the upper right quadrant, where elevated IRR coincides with favorable production cost structures. Although small localized blue pockets appear within this red-dominant region, they do not represent distinct, feasible optima. Rather, they arise from localized nonlinear interactions captured by the XGBoost model and from smoothing effects associated with the cubic interpolation of discrete simulation outputs. These minor variations reflect local surface fluctuations rather than economically stable regimes.

Conversely, the lower left quadrant, characterized by low IRR and low MPSP, represents economically unfavorable conditions. Transitional regions between these extremes illustrate the sensitivity of financial performance to variations in both IRR and UPC.

To further evaluate MPSP variability under different financial conditions, kernel density estimation (KDE) was integrated into the contour visualization. This method enhances the identification of dense and stable economic regions while highlighting areas more sensitive to parameter fluctuations. The analysis confirms that although cost efficiency contributes to profitability, IRR exerts the strongest influence on MPSP across the evaluated domain.

Figure 8 provides valuable insights into profitability trends by illustrating the interaction between the IRR (after taxes) and UPC in determining the MPSP.

Because MPSP is directly linked to the minimum attractive rate of return (MARR), a 30% MARR threshold was adopted to reflect the risk profile associated with emerging technologies. Investment feasibility was therefore assessed by comparing IRR values to this benchmark: projects with IRR ≥ 30% were considered economically attractive.

Regions meeting this criterion are concentrated in the upper portion of the heatmap, particularly within warmer-colored zones that indicate stronger revenue performance. To quantify feasible pricing conditions, the analysis focused on the region where UPC ranges between USD 140/kg and USD 240/kg. Within this interval and under the IRR ≥ 30% constraint, the corresponding MPSP values extracted from the heatmap range between USD 262/kg and USD 525/kg.

Strategic positioning within the upper right sector of the heatmap, where IRR remains above the 30% threshold and revenue is maximized, is essential for ensuring long-term economic viability. Maintaining profitability requires continuous control of production costs and careful pricing strategies, as increases in UPC or reductions in MPSP may lower the IRR below the acceptable investment threshold. Periodic scenario analysis is therefore recommended to preserve financial resilience under the evolving market conditions.

## 4 Conclusion

This study presents an ML-based framework for stochastic TEA in API production. By integration of ML with process simulation, the framework enables the development of predictive models for estimating the MPSP and UPC, significantly reducing the reliance on iterative simulation.

The approach offers a scalable and automated alternative to traditional TEA, improving cost estimation, sensitivity analysis, and risk assessment in pharmaceutical manufacturing. Among the evaluated models, XGBoost achieved the highest predictive performance, and its evaluation on the hold-out test set showed comparable results, confirming the model’s strong generalization capability beyond the training data. LASSO regression was used to identify key economic drivers, revealing that financial parameters (e.g., IRR, loan period, and income taxes) strongly influence the MPSP, while raw material and labor costs are the primary contributors to the UPC. Dimensionality reduction further enhanced model performance by optimizing input variable selection.

By combining KDE and SHAP analyses, this study identifies the key drivers of MPSP and UPC uncertainty, showing that skewed investment- and tax-related variables propagate greater prediction variability, while operational costs with stable distributions provide consistent predictive value, supporting more robust financial and operational planning.

Overall, the findings underscore the value of ML-driven TEA in enhancing the efficiency, accuracy, and applicability of economic evaluations in the pharmaceutical manufacturing. Future research could focus on the development of dynamic pricing models and real-time optimization strategies, extending this framework to broader applications in pharmaceutical, chemical, and biochemical sectors.

## Supplementary Material



## Notes

Additional tables: equipment purchase costs; raw material consumption in the API production process; main parameters and values considered in the simulation baseline of the active pharmaceutical ingredient (API); total capital investment (CAPEX) considered for the API production facility; breakdown of the annual operating expenditure (OPEX) per process stage; feature importance scores for the nine variables identified as most relevant for MPSP and UPC prediction after LASSO feature selection. Additional figures: Pearson correlation matrix of all variables in the data set; predicted versus actual UPC values across different ML models after feature selection (PDF)

## List of abbreviations

AI: artificial intelligence
ANN: artificial neural networks
APIs: active pharmaceutical ingredients
ARIMA: auto regressive integrated moving average
BRICS: Brazil, Russia, India, China, South Africa, Egypt, Ethiopia, Indonesia, Iran, and the United Arab Emirates
CAPEX: capital expenditures
COM: microsoft component object model
GPU: graphics processing units
IRR: internal rate of return
KDE: Kernel density estimation
KNN: K-nearest neighbors
LASSO: least absolute shrinkage and selection operator
LR: linear regression
LSTM: long short-term memory
MAPE: mean absolute percentage error
MARR: minimum attractive rate of return
MINT: Mexico, Indonesia, Nigeria, and Turkey
ML: machine learning
MPSP: minimum product selling price
OPEX: operational expenditures
*R* ^2^: *R*-squared (coefficient of determination)
RMSE: root mean squared error
SHAP: shapley additive explanations
SVM: support vector machines
TEA: techno-economic assessment
TPU: tensor processing units
UPC: unit production cost
XGBoost: extreme gradient boosting
