# Supplementary material for: Machine Learning-Driven Techno-Economic Uncertainty Analysis in Batch Pharmaceutical Manufacturing
Source: ACS Omega. 2026 May 21;11(21):30431–47. doi: 10.1021/acsomega.5c09455 (PMC13234674; doi:10.1021/acsomega.5c09455)
Supplement: Supplementary file 1 [file ao5c09455_si_001.pdf]

Supporting Information

Machine Learning-Driven Techno-Economic Uncertainty Analysis in Batch Pharmaceutical Manufacturing

[1]Diego Andres Rueda Ordonez, [1]Letícia Costa da Silva Mesquita, [1*]Amanda Lemette Teixeira Brandão

[1]Department of Chemical and Materials Engineering, Pontifical Catholic University of Rio de Janeiro, 225 Marquês de São Vicente Street, Gávea, 22451900, Rio de Janeiro, RJ – Brasil

*Corresponding Author: amanda.lemette@puc-rio.br

Supporting Information Available: Additional tables: equipment purchase costs; raw material consumption in the API production process; main parameters and values considered in the simulation baseline of the active pharmaceutical ingredient (API); total capital investment (CAPEX) considered for the API production facility; breakdown of the annual operating expenditure (OPEX) per process stage; feature importance scores for the nine variables identified as most relevant for MPSP and UPC prediction after LASSO feature selection. Additional figures: Pearson correlation matrix of all variables in the dataset; predicted versus actual UPC values across different ML models after feature selection.

Table S1. Equipment purchase cost

Main Equipment		
Name	Description	Unit Cost (USD)
NFD-101	Nutsche Filter Filter Area = 2,50 m2	161,000
NFD-102	Nutsche Filter Filter Area = 2,50 m2	161,000
R-101	Stirred Reactor Vessel Volume = 3780,00 L	111,000
R-102	Stirred Reactor Vessel Volume = 3780,00 L	111,000
R-103	Stirred Reactor Vessel Volume = 3780,00 L	111,000
SB-101	Solids Bin Vessel Volume = 307,50 L	94,000
T-101	Blending Tank Vessel Volume = 1889,54 L	66,000
TDR-101	Tray Dryer Drying Area = 10,00 m2	69,000
	Unlisted Equipment	221,000
	TOTAL	1,105,000

Table S2. Raw Material consumption in the APIs production process

Material	kg/yr	kg/batch	kg/kg APIs
Carbon tetrachloride	94,650	706.344	2.871
Chlorine	17,037	127.142	0.517
HCl (20% w/w)	68,030	507.685	2.064
Hydroquinone	32,631	243.512	0.99
Methanol	105,298	785.807	3.194
Na2CO3	19,995	149.215	0.607
NaOH (50% w/w)	38,925	290.484	1.181
Quinaldine	28,289	211.108	0.858
Water	143,288	1,069.32	4.347
**Isolation and Purification**			
**Material**	**kg/yr**	**kg/batch**	**kg/kg APIs**
Sodium Hydroxide	14,115	105.334	0.428
Water	581,864	4,342.27	17.651
**Final Purification**			
**Material**	**kg/yr**	**kg/batch**	**kg/kg APIs**
charcoal	3,017	22.515	0.092
HCl (37% w/w)	41,409	309.025	1.256
Isopropanol	330,795	2,468.62	10.035
Water	213,601	1,594.03	6.48
**Crystallization and Drying**			
**Material**	**kg/yr**	**kg/batch**	**kg/kg APIs**
Isopropanol	94,060	701.943	2.853
Nitrogen	211,487	1,578.26	6.416

Table S3. Main parameters and values considered in the simulation baseline of the active pharmaceutical ingredient (API)

**Materials Cost**				
		Annual Amount (kg)	Annual Cost (USD)	%
Quinaldine cost	32.0	94,650.0	75,720.0	3.6
Isopropanol cost	1.1	17,037.0	56,222.0	2.7
Nitrogen cost	1.0	32,631.0	130,522.0	6.2
Na2CO3 cost	6.5	424,855.0	467,341.0	22.3
Hydroquinone cost	4.0	19,995.0	129,966.0	6.2
Carbon tetrachloride cost	0.8	211,487.0	211,487.0	10.1
Chlorine cost	3.0	28,289.0	905,233.0	43.1
Sodium Hydroxide cost	2.0	14,115.0	28,229.0	1.3
Water for the process	0.1	143,288.0	14,329.0	0.7
Wash water cost	0.1	795,465.0	79,546.0	3.8
			2,098,595.0	100.0
**Waste Treatment**				
		Annual Amount (kg)	Annual Cost (USD)	%
Carbon tetrachloride Waste cost	5.0	94,650.0	473,249.8	19.92
CO2 Waste cost	5.0	8,011.7	40,058.6	1.69
Isopropanol Waste cost	2.0	178,374.4	891,871.8	37.53
Sodium Hydroxide Waste cost	2.0	8.3	16.6	0.00
Charcoal Waste cost	2.0	3,016.9	6,033.8	0.25
Quinaldine Waste cost	2.0	565.5	1,131.0	0.05
Chloroquinaldine Waste cost	2.0	750.4	1,500.8	0.06
Product Waste cost	2.0	1,132.0	2,264.1	0.10
Product crystal Waste cost	2.0	3,660.3	7,320.7	0.31
Impurity Waste cost	2.0	635.4	1,270.9	0.05
Product.Na Waste cost	2.0	50.7	101.3	0.00
Chlorine Waste cost	2.0	3,308.2	16,541.0	0.70
HCL Waste cost	2.0	2,469.9	12,349.4	0.52
Hydroquinone.Na Waste cost	2.0	14,223.3	71,116.5	2.99
Methanol Waste cost	2.0	105,298.1	526,490.7	22.16
Na2CO3 Waste cost	2.0	28.1	140.7	0.01
Hydroquinone Waste cost	2.0	130.4	651.9	0.03
NaCL Waste cost	2.0	64,789.0	323,945.0	13.63
			2,376,055	100.00
**Labor operations**				
	Unit Cost (USD/h)	Annual Amount (h)	Annual Cost (USD)	
Operator	41.4	29,901	1,237,918	

Financial variables			
	Unit	Value	Annual Cost (USD)
Income Taxes	%	40	4,282,000
Loan Interest	%	9	702,810
Loan Period	years	10	-
Annual Operation time (h)	hours	7,920.00	-
Inflation	%	4	-

Table S4. Total capital investment considered for the active pharmaceutical ingredient (API) production facility (CAPEX)

**Total Capital Investment**	
**Total Plant Direct Cost (TPDC) (physical cost)**	USD
1. Equipment Purchase Cost	1,105,000
2. Installation	658,000
3. Process Piping	387,000
4. Instrumentation	442,000
5. Insulation	33,000
6. Electrical	111,000
7. Buildings	497,000
8. Yard Improvement	166,000
9. Auxiliary Facilities	442,000
**TPDC**	**3,841,000**
**Total Plant Indirect Cost (TPIC)**	
10. Engineering	960,000
11. Construction	1,344,000
**TPIC**	**2,304,000**
**Total Plant Cost (TPC = TPDC+TPIC)**	
**TPC**	**6,145,000**
**Contractor's Fee & Contingency (CFC)**	
12. Contractor's Fee	307,000
13. Contingency	614,000
**CFC = 12+13**	**921,000**
**Direct Fixed Capital Cost (DFC)**	
**DFC = TPC+CFC**	**7,066,000**

Table S5. Breakdown of the annual OPEX of the API production per process stage

COST PER PROCESS SECTION			
Product Synthesis (Stage 1)	USD/kg MP	USD/batch	USD/year
Raw Materials	40.7	10,023.0	1343103.0
Facility-Dependent	5.2	1,284.0	172094.0
Labor-Dependent	8.9	2,190.0	293399.0
Laboratory/QC/QA	1.3	328.0	44010.0
Waste Treatment/Disposal	6.9	1,691.0	226567.0
Total	63.1	15,516.0	2079173.0
Isolation and Purification (Stage 2)	USD/kg MP	USD/batch	USD/year
Raw Materials	2.6	645.0	86,416.0
Facility-Dependent	6.4	1,579.0	211,640.0
Labor-Dependent	8.1	1,992.0	266,970.0
Laboratory/QC/QA	1.2	299.0	40,045.0
Waste Treatment/Disposal	0.2	40.0	5,296.0
Total	18.5	4,555.0	610,367.0
Final Purification (Stage 3)	USD/kg MP	USD/batch	USD/year
Raw Materials	12.1	2,977.0	398,912.0
Facility-Dependent	13.8	3,400.0	455,545.0
Labor-Dependent	9.7	2,397.0	321,155.0
Laboratory/QC/QA	1.5	360.0	48,173.0
Waste Treatment/Disposal	0.3	74.0	9,931.0
Total	37.4	9208.0	1,233,716.0
Crystallization and Drying (Stage 4)	USD/kg MP	USD/batch	USD/year
Raw Materials	9.6	2,350.0	314,953.0
Facility-Dependent	14.7	3,624.0	485,682.0
Labor-Dependent	10.8	2,660.0	356,395.0
Laboratory/QC/QA	1.6	399.0	53,459.0
Waste Treatment/Disposal	19.5	4,791.0	641,978.0
Total	56.2	13,824.0	1,852,467.0

Table S6. Feature importance scores for the nine variables identified as most relevant to MPSP and UPC prediction after LASSO feature selection.

Feature	Importance
IRR	499.40
Loan period	132.61
Labor (USD/h)	83.13
Quinaldine (USD/kg)	67.62
Income taxes	40.71
Isopropanol (USD/kg)	37.29
Water (USD/kg)	37.04
Annual operation time (h)	29.70
Nitrogen (USD/kg)	29.08

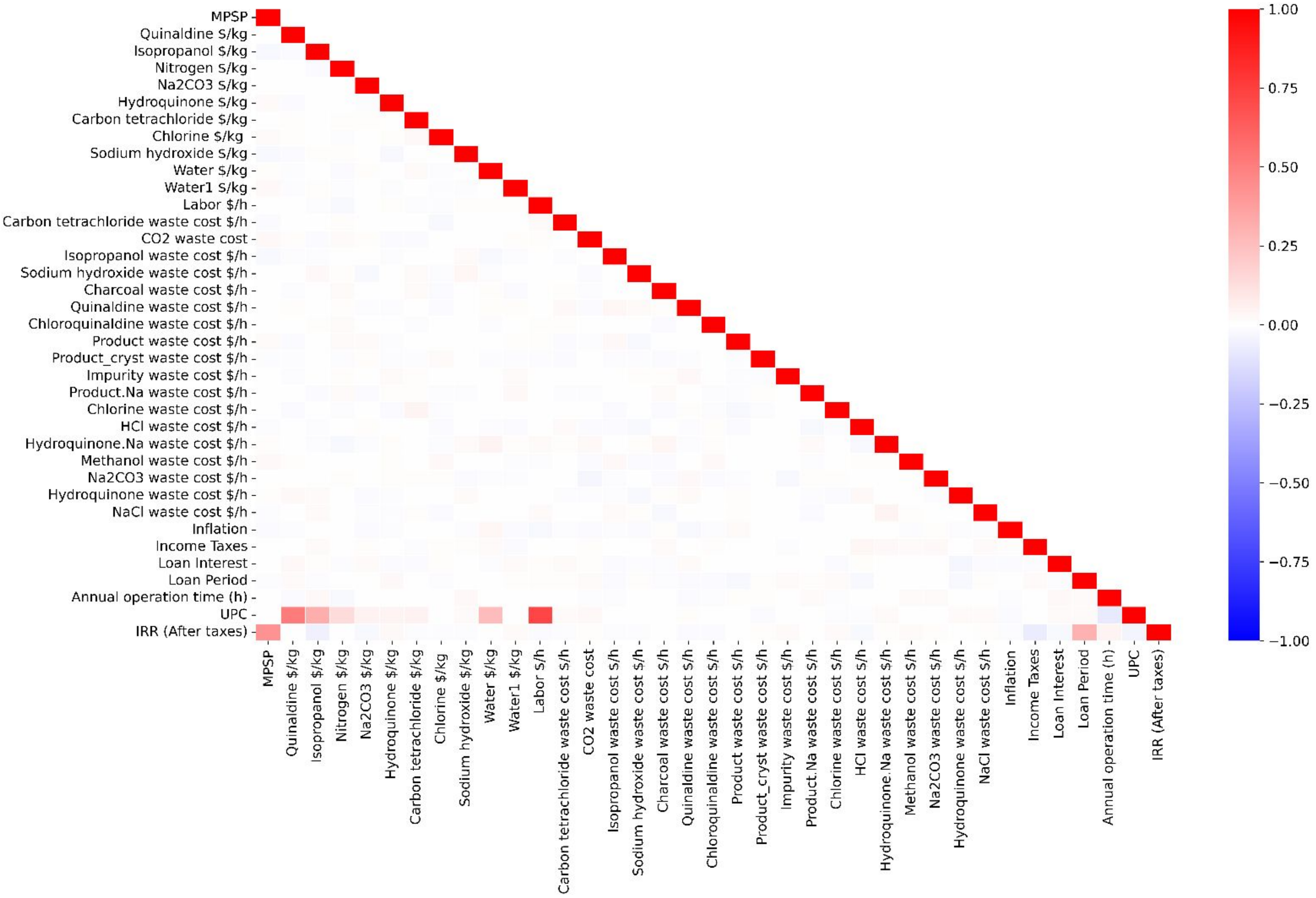

Figure S1. Pearson correlation matrix of all variables in the dataset.

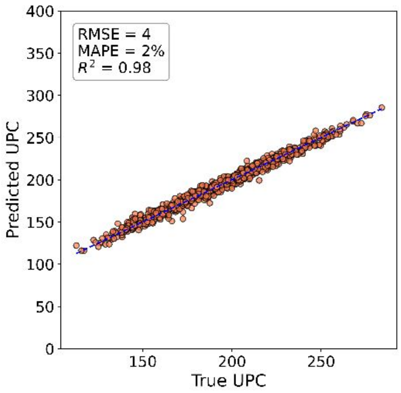

(a) XGBoost

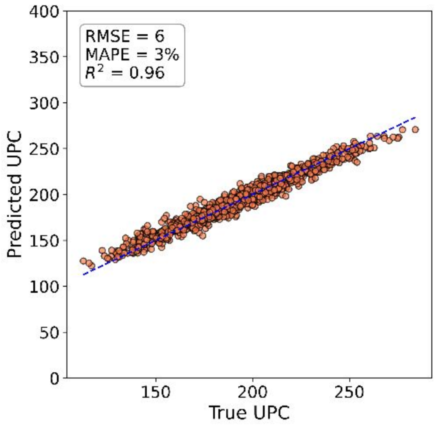

(b) Random Forest

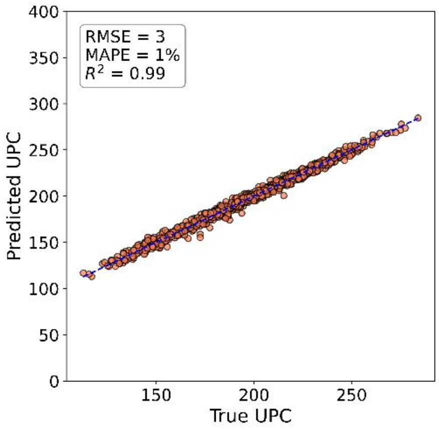

(c) Polynomial ridge

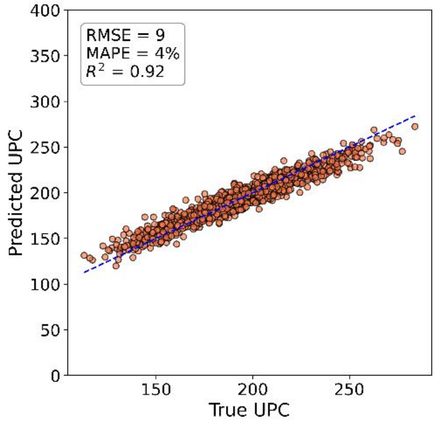

(d) KNN

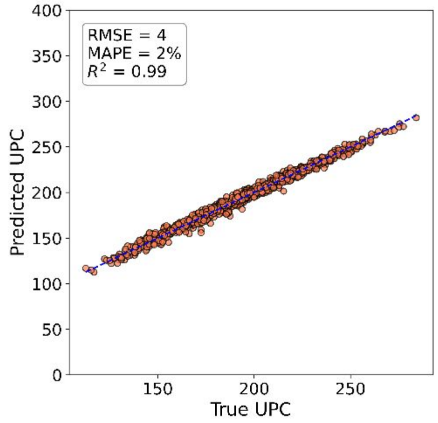

(e) Linear regression

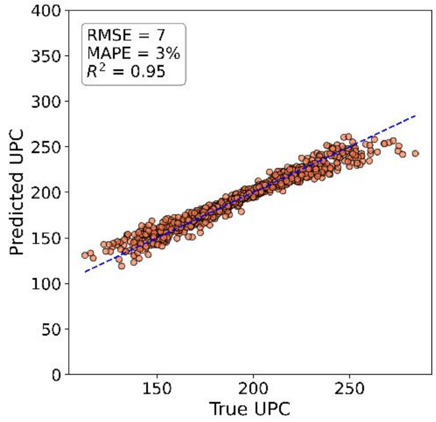

(f) SVR

Figure S2. Predicted vs. actual values for UPC across different ML models after the execution of the feature selection.
